# Polyribosomal RNA-Seq Reveals the Decreased Complexity and Diversity of the Arabidopsis Translatome

**DOI:** 10.1371/journal.pone.0117699

**Published:** 2015-02-23

**Authors:** Xingtan Zhang, Benjamin D. Rosen, Haibao Tang, Vivek Krishnakumar, Christopher D. Town

**Affiliations:** 1 J. Craig Venter Institute, Rockville, Maryland, United States of America; 2 School of Life Sciences, Chongqing University, Chongqing, China; University of Toronto, CANADA

## Abstract

Recent RNA-seq studies reveal that the transcriptomes in animals and plants are more complex than previously thought, leading to the inclusion of many more splice isoforms in annotated genomes. However, it is possible that a significant proportion of the transcripts are spurious isoforms that do not contribute to functional proteins. One of the current hypotheses is that commonly used mRNA extraction methods isolate both pre-mature (nuclear) mRNA and mature (cytoplasmic) mRNA, and these incompletely spliced pre-mature mRNAs may contribute to a large proportion of these spurious transcripts. To investigate this, we compared a traditional RNA-seq dataset (total RNA-seq) and a ribosome-bound RNA-seq dataset (polyribosomal RNA-seq) from *Arabidopsis thaliana*. An integrative framework that combined *de novo* assembly and genome-guided assembly was applied to reconstruct transcriptomes for the two datasets. Up to 44.8% of the *de novo* assembled transcripts in total RNA-seq sample were of low abundance, whereas only 0.09% in polyribosomal RNA-seq *de novo* assembly were of low abundance. The final round of assembly using PASA (Program to Assemble Spliced Alignments) resulted in more transcript assemblies in the total RNA-seq than those in polyribosomal sample. Comparison of alternative splicing (AS) patterns between total and polyribosomal RNA-seq showed a significant difference (G-test, p-value<0.01) in intron retention events: 46.4% of AS events in the total sample were intron retention, whereas only 23.5% showed evidence of intron retention in the polyribosomal sample. It is likely that a large proportion of retained introns in total RNA-seq result from incompletely spliced pre-mature mRNA. Overall, this study demonstrated that polyribosomal RNA-seq technology decreased the complexity and diversity of the coding transcriptome by eliminating pre-mature mRNAs, especially those of low abundance.

## Introduction

As a result of the development of deep sequencing technology, our understanding of transcriptome complexity has been greatly improved during the past decade. An example of this improvement is alternative splicing analysis in plants. The first wave of genome-wide transcriptome analysis consisted of direct sequencing of cDNAs and ESTs on a large scale and suggested that 11 to 30% of the multi-exonic genes in plants were alternatively spliced [1,2]. Afterwards, deeper sequencing provided by RNA-seq technology led to the observation that up to 60% of intron-containing genes in Arabidopsis could be alternatively spliced under various conditions [3,4]. However, the dramatically increased identification of alternative splicing events raises doubts as to their biological relevance. Are these alternate splice isoforms functional, thus justifying their inclusion in the genome annotation or are they mostly the result of random background transcription (noise) or splicing errors?

In many cases, RNA-seq libraries are constructed from total cellular RNA, which is a mixture of nuclear and cytoplasmic RNA and contains, in addition to protein-coding RNA, a variety of non-coding RNAs. In the nucleus, protein-coding RNAs are capped, polyadenylated and spliced before exiting to the cytoplasm as mature mRNAs. While some coding RNAs may be retained in the nucleus awaiting specific environmental or developmental cues, much of the nuclear RNA population consists of partially processed transcripts in which intron splicing is incomplete. In a total RNA-seq analysis, these incompletely spliced isoforms can contribute additional but potentially misleading variants of the annotated gene structures.

Increasing evidence shows that a proportion of transcript isoforms, whether due to incomplete processing or spurious splicing of background transcripts may not contribute to the functional protein pool, likely due to nonsense-mediated mRNA decay (NMD) [5,6]. NMD is a translation-coupled mechanism that eliminates mRNAs containing premature termination codons (PTCs). Analysis of alternative splicing in humans revealed that one-third of alternative transcripts contained premature termination codons based on EST evidence [5]. Similar results were observed in plants. Based on RNA-seq data, it was estimated that up to 70% of predicted AS transcripts introduced in-frame PTCs in Arabidopsis [6]. These PTCs are located more than 55 nt upstream of an exon-intron junction, a situation that was found in *Nicotiana benthamiana* cells to correlate with the likelihood that they would be subject to NMD [7], hence the “55 nt rule”. In addition to NMD, it has been proposed that most low abundance alternative isoforms are likely due to splicing errors for two reasons: i) those rarely-used splice sites in low-abundance isoforms are enriched near often-used splice sites and show a periodic pattern around those sites; ii) these rarely-used splice sites are less evolutionary conserved [8].

Another obvious yet less-discussed reason for spurious/low abundance isoforms is that in addition to various species of non-coding RNAs and non-specific background transcription, total cellular RNA isolated by current methods (which often include polyA+ selection) contains a mixture of pre-mature and mature mRNAs. Sequencing of such mixtures can lead to the reconstruction of incompletely spliced isoforms and the annotation of non-existent gene models. It can also lead to a lack of correlation between mRNA and protein levels [9]. To circumvent this problem, researchers began to focus on ribosome-associated RNA for their studies: the translatome. This was facilitated by the development of plants expressing an epitope-tagged ribosomal protein allowing the recovery of ribosome-bound mRNAs that were used to study the recruitment of mRNAs to ribosomes in response to environmental cues such as stress [10]. With the advent of RNA-seq, this isolation method was used for a number of studies including measurement of the abundance of mature mRNAs that are associated with the process of translation and identification of sites of ribosome pausing [11]. The technology provides a more accurate strategy to measure gene expression at the level of translation. For example, *PERK10* (PROLINE-RICH EXTENSIN-LIKE RECEPTOR KINASE 10) encodes a proline-rich protein and the translational efficiency of its mRNA is increased during hypoxia based on ribosome-profiling evidence; however, sequencing of total mRNA extracts failed to detect this change [12]. Application of this technology also resulted in other impressive outcomes [9]. For instance, investigation of genome-wide regulation of translation in response to heat shock using ribosome profiling reveals that heat shock induces an arrest at around codon 65 on most mRNAs in both mouse and human cells [13].

Despite its merit, one of the limitations of ribosome profiling technology is that it is particularly challenging to assemble complete transcripts from the short fragments isolated for sequencing, and it is therefore impossible to comprehensively identify alternative isoforms and monitor the translational dynamics of splicing in cells [9]. RNA-blot analysis showed that immunopurification of polyribosomes (a mixture of ribosomes from 1 to 5 or more ribosomes [14]) yielded intact mRNAs, suggesting a potential application to explore the pattern of polyribosome-associated alternatively spliced mRNAs [14]. RNA-seq of polyribosomal RNA from *Medicago truncatula* successfully demonstrated that differential translation of mRNAs significantly contributed to the regulation of gene expression during nodule formation [15]. Recent research on the Arabidopsis bundle sheath (BS) transcriptome combined the polyribosomal RNA extraction method and RNA-seq technology to investigate specific expression and splicing patterns in BS cells [16]. Although the polyribosomal RNA technology is a powerful tool for interpreting post-transcriptional regulation of gene expression, the differential alternative splicing patterns between polyribosomal mRNA-seq and total mRNA-seq are far from well explored on a genome-wide basis and are the subject of this paper.

In our study, we applied a combination of *de novo* and genome-guided assembly methods to reconstruct the transcriptomes for total RNA-seq and polyribosomal RNA-seq samples. Comparison of alternative splicing patterns between the two assembled data sets revealed a significant difference in intron retention. Our results reveal that polyribosomal RNA-seq decreased the complexity and diversity of transcriptome. We demonstrate here that polyribosomal RNA-seq technology is an efficient method to eliminate some pre-mature mRNAs likely resulting from incomplete splicing.

## Results and Discussion

### Data processing

We compared the splicing patterns using RNA-seq data derived from 3–4 weeks old Col-0 leaves. The polyribosomal data consisted of a total of nearly 96 million reads (101-nt read length; 5.8 GB single-end Illumina data for whole leaves) [16]. Since traditional total mRNA sequencing was not carried out in this polyribosome-capture study, we identified a comparable set of total RNA-seq data in the NCBI short read archive (SRX152606). In this second study, total RNA was used to construct a polyadenylated leaf RNA library from 2–3 weeks old Col-0 leaves and sequenced using Illumina HiSeq 2000 with 101-cycle single-end sequencing protocol [17]. Since the two datasets have the same read-length, we randomly extracted the same numbers of reads (95.5 million for each) from the two datasets to achieve a nonbiased analysis (S1 Table).

**Fig 1:**
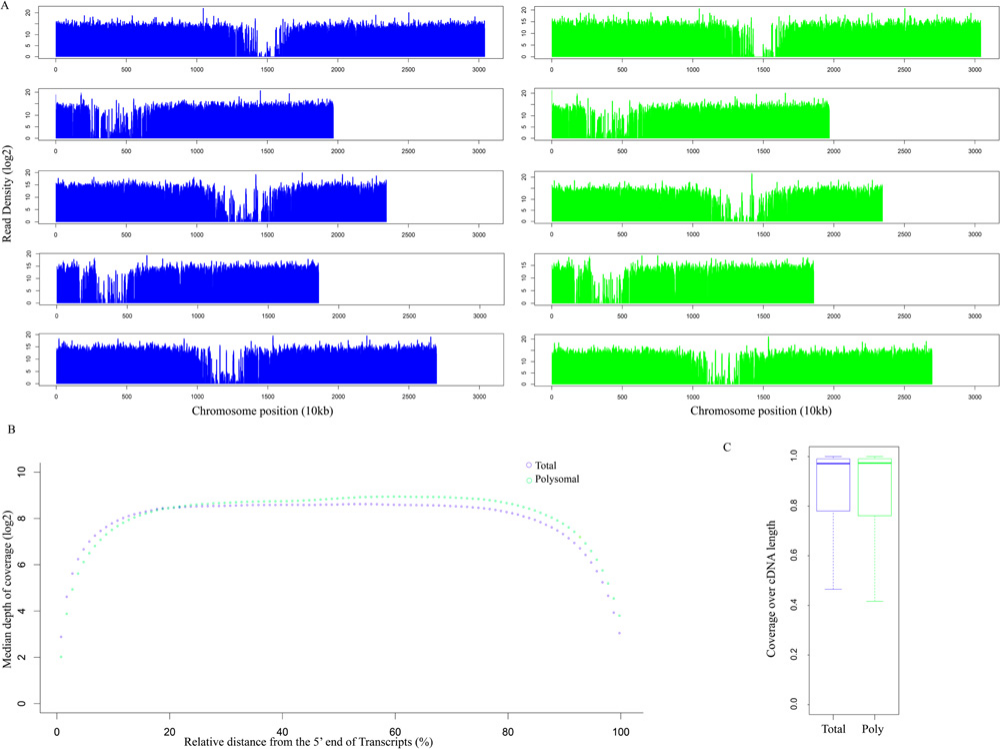
Coverage profiles along *A*. *thaliana* chromosomes and TAIR10 annotated CDS. (A) Distribution of RNA-seq read density along chromosome length is shown for total RNA-seq (left) and polyribosomal RNA-seq (right). The y axis represents the log2 scale of median read density. (B) Distribution of the RNA-seq read coverage along the length of the transcriptional unit. The log2 scale of median depth of coverage along the length of each individual TAIR10 annotated cDNA was calculated and plotted against the relative length of the transcriptional unit for the total RNA-seq and polyribosomal RNA-seq. (C) Coverage over the length of TAIR10 annotated CDS. Box-and-whisker plots depict the coverage calculated as the percentage of bases along the length of the cDNA sequence that was supported by reads from the total and polyribosomal RNA-seq datasets. The bottom and top of the boxes represent the 25^th^ and 75^th^ quartiles, respectively. The lines within boxes represent the medians.

To investigate whether polyribosomal RNA-seq is a nonbiased method for a broad survey of the transcriptome, we aligned total RNA-seq and polyribosomal RNA-seq reads to the Arabidopsis genome (TAIR10) using Tophat v2.0.8 [18] and compared coverage profiles along 5 chromosomes for the two datasets (Fig. 1A). 95.2% of total RNA-seq and 96.5% of polyribosomal RNA-seq reads were mapped to the genome. Comparison of coverage profiles revealed that total RNA-seq and polyribosomal RNA-seq have similar chromosome coverage patterns. In addition, we compared the depth of coverage along the length of individual transcriptional units (Fig. 1B). Our results revealed that both total RNA-seq and polyribosomal RNA-seq were evenly distributed. Analysis of the RNA-seq coverage over the lengths of all TAIR10 annotated cDNAs demonstrated that the coverage profiles were similar in the two types of libraries (Fig. 1C), and overall 50% of TAIR10 annotated cDNAs had at least 97% of their sequence lengths represented by both of the samples. These coverage results suggest that the trimmed total and polyribosomal RNA data kept the integrity of transcriptome, providing the possibility to survey whole-genome wide alternative splicing patterns.

### Reconstruction of the Arabidopsis transcriptome reveals decreased complexity in polyribosomal RNA-seq

To obtain an accurate and comprehensive transcriptome database, we performed both a *de novo* as well as a genome-guided assembly, and then combined the outputs using the PASA pipeline [19]. The advantages of this approach are apparent [20]. First, the Arabidopsis genome and annotation have been well developed and provide a good guidance to improve transcriptome reconstruction. Therefore, a genome-guided strategy is very sensitive and can assemble transcripts of low abundance. In addition, contamination or sequencing artifacts are not a major concern for this strategy, because these reads are not expected to align to the reference genome. Second, *de novo* assembly is still popular even when the reference genome is available, as it can recover transcripts that are derived from segments of the genome that are missing from the genome assembly. It can also detect transcripts from an unknown exogenous source. The PASA pipeline is then used to take advantage of the high sensitivity of reference-based assembly while leveraging the ability of *de novo* assembly to detect novel transcripts [1].

The Trinity *de novo* assembly resulted in 42,962 and 35,545 transcripts for total RNA-seq and polyribosomal RNA-seq samples respectively (Table 1). To avoid low-abundance (noisy) isoforms and likely transcript artifacts [8], we only retained isoforms that represented at least 3% of the expression level of that locus and had an FPKM value of at least 1. These criteria were based on inspection of many alternately spliced gene loci in JBrowse. The filtering step led to a surprising result: 44.8% of transcripts in the total RNA-seq *de novo* assembly were discarded; however, only 0.09% of *de novo* assembled transcripts in polyribosomal RNA-seq were filtered. The genome-guided assembly did not show such big difference (1.4% of total RNA-seq and 0.6% of polyribosomal RNA-seq transcripts were dropped). This result revealed that *de novo* assembly for total RNA-seq generated more low abundance transcripts, whereas polyribsomal RNA-seq seemed to be consistent before and after filtering. Finally, we used PASA to integrate the *de novo* assembly and genome-guided assembly, generating comprehensive transcript databases (34,231 transcripts for total RNA-seq and 26,959 transcripts for polyribosomal RNA-seq) (Table 1).

**Table 1:** Assembly of total RNA-seq and polyribosomal RNA-seq and AS estimates.

	No. of Assembled transcripts		No. of transcripts after filtering		PASA Assembly
	De novo	Genome-guided	De novo	Genome-guided	
Total RNA	42,962	38,340	23,697	37,785	34,231
Poly RNA	35,545	29,461	35,259	29,298	26,959
		Alternative splicing estimates			
		Total RNA sample		Polyribosomal RNA sample	
Genes detected in samples		12,876		12,515	
Intron-containing genes		11,967		11,571	
Alternatively Spliced genes		2,090		1,396	
% of genes with AS		17.5%		12.1%	

To evaluate whether the sets of comprehensive assembled transcripts correspond to known isoforms in TAIR10, we calculated how many genes with assembled transcripts had at least one isoform that resembled an annotated transcript in TAIR10. We required the assembled transcript to contiguously cover at least 80% of the total length of the annotated isoform and have at least 95% identity with TAIR10 cDNAs. We found that 50% of genes within total RNA-seq assembled transcript set and 45.6% of genes within the polyribosomal RNA-seq assembled transcript set had at least one transcript that is annotated in TAIR10 (Table 1).

Previous research on Arabidopsis using RNA-seq showed that up to ~61% of genes with introns had evidence of alternative splicing [3]. To estimate the numbers of intron-containing genes with AS for the total and polyribosomal samples, we calculate the ratios of genes with more than one putative transcript divided by the numbers of intron-containing genes in each sample. The total and polyribosomal samples show similar results: 17.5% of genes with introns in total RNA-seq sample and 12.1% in polyribosomal sample had more than one isoform (Table 1). Several reasons can explain why we identified the lower AS rates in this study comparing to the previous publication [3]. It is possible that the previously published figure of AS rate consist of transcripts that do not survive our filtering steps. To investigate this, we constructed comprehensive database for the total RNA-seq using the pre-filtered Trinity *de novo* and genome-guided assembled transcripts. The number of intron-containing genes showing AS in the unfiltered transcript set (19.4%; 2,379/12,247) is only slightly higher than that observed after filtration. A more likely explanation is that the previous study analyzed mixed RNA isolated from flowers under different stages combined with 10-d-old seedlings, whereas we only identified AS in leaves. Therefore, it is expected that more alternative splicing events can be found in their study. Moreover, the previous study used Tophat to identify AS events, whereas we applied PASA. The PASA algorithm is based on alignment assembly, which is very stringent in its detection of alternative isoforms [21].

It is reported that more than one-third of alternatively spliced isoforms harbored pre-mature termination codons (PTCs) in human and Arabidopsis [5,6,22]. These transcripts with PTCs are potential targets of nonsense-mediated mRNA decay (NMD), a surveillance mechanism that selectively degrades nonsense mRNAs. Previous work in plants has shown that PTCs located 55 nt or more upstream from an exon-intron boundary are likely candidates for NMD (hence the 55 nt rule) [7]. In the total and polyribosomal RNA-seq samples analyzed here, 34.4% of alternatively spliced transcripts in the total sample and 39.4% in the polyribosomal sample introduced in-frame PTCs that resided more than 55 nt upstream of an exon/exon junction, consistent with previous results [5,6,22].

### Polyribosomal RNA-seq reveals a decreased diversity in mRNA isoforms

Traditional mRNA extraction has the potential to mix RNA molecules from the cell nucleus and cytoplasm, generating a mixture of pre-mature mRNA and mature mRNA. It is likely that pre-mature mRNA contribute to an overestimated prediction of alternative splicing events. The recently developed polyribosomal RNA extraction technology has provided a new strategy to obtain polyribosome-bound mRNA, most of which are assumed to be mature mRNA [14]. Thus, it is expected that total RNA sample and polyribosomal RNA sample may show different patterns of alternative splicing.

**Fig 2:**
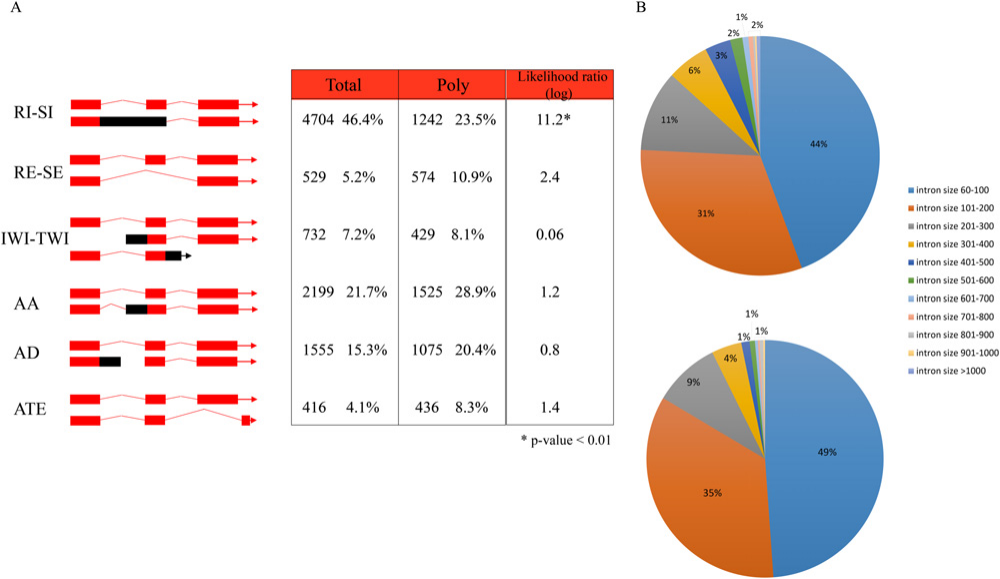
Different patterns of alternative splicing between total RNA-seq and polyribosomal RNA-seq. (A) Six classes of alternative splicing events in the two samples. RI-SI: retained intron or skipped intron. RE-SE: retained exon and skipped exon. IWI-TWI: initiation within intron or termination within intron. AA: alternative acceptor. AD: alternative donor. ATE: alternative terminal exon. G-test was used to calculate likelihood ratio statistics. (B) Distribution of retained intron sizes predicted by PASA in total RNA-seq (top circle) and polyribosomal RNA-seq (bottom circle).

To investigate the different patterns of alternative splicing, we employed PASA [1] to identify the alternative splicing events. PASA defines nine classes of alternative splicing: a) Alternate Acceptor (AA), b) Alternate Donor (AD), c) Alternate Terminal Exon (ATE), d) Skipped Exon (SE), e) Retained Exon (RE), f) Initiation Within an intron (IWI), g) Termination Within an Intron (TWI), h) Retained Intron (RI), and i) Spliced Intron (SI). The RE and SE classes are reciprocal meaning that an assembly of the SE class is complemented by its cognate paired assembly displaying the RE class. Likewise, the SI and RI classes are reciprocal. In addition, the IWI and TWI classes are conceptually similar where either the 5′ or the 3′ end of the transcript isoform occurs in an intron of its longer isoform. Therefore, we re-assigned the AS events into six categories: AA, AD, ATE, RI/SI, SE/RE and IWI/TWI (Fig. 2A).

Overall, 10,135 and 5,281 alternative splicing events were identified, affecting 2,090 and 1,396 genes in total RNA and polyribosomal RNA samples, respectively. This marked decrease from the total to the polyribosomal sample suggests that the polyribosomal transcriptome is less complex and diverse. Prior research on Arabidopsis alternative splicing either based on cDNAs/ESTs or total RNA-seq reported that intron retention was the predominant type of alternative splicing in Arabidopsis [3,23]. Our result for total mRNA-seq reveals the same pattern. 46.4% of all AS events are RI/SI, which is the major AS type in total RNA-seq. By contrast, only 23.5% of AS events in polyribosomal RNA are assigned to RI/SI, which is significantly different from RI/SI events in total RNA-seq (G-test, p-value<0.001). In the total RNA sample 38% (1,786/4,704) of the unspliced introns contained stop codons whereas in the polyribosomal sample the corresponding number was 24% (303/1242). This indicates that the majority of retained introns in mRNA undergoing translation can be read through and contribute to the mature protein sequence. RI/SI events constitute the second most abundant class in the polyribosomal sample with alternate acceptor (AA) being the predominant one, with 28.9% of AS events compared to 21.7% in total RNA. The other four classes reveal similar proportions between total and polyribosomal RNA (Fig. 2A).

It is reported that intron size is one of the factors that impacts splicing efficiency [3]. In the TAIR10 protein-coding genes, the average and median sizes of all introns are 165 nt and 99 nt, respectively. We also investigated the sizes of retained introns for the two samples (Fig. 2B). Similar to previous research [3], the majority (75% in total; 84% in polyribosomal) of retained-intron size were smaller than 200 nt. The retained introns in the total RNA-seq sample had a mean size of 171 nt (median = 108), whereas those in polyribosomal sample were smaller (mean = 143 nt versus mean = 171 nt, Mann-Whitney-Wilcoxon test, p-value = 3.627 x 10^–6^). These results suggest that the longer introns tend to be spliced during pre-mRNA processing.

In conclusion, this analysis revealed that many isoforms with intron retention AS events in total RNA-seq are not found in the polyribosomal fraction. Many of these are likely to be incompletely processed mRNAs. However, other scenarios should not be overlooked. Some primary mRNAs may be retained in the nucleus awaiting processing and transit to the cytosol upon a developmental or environmental cue. Other mRNAs may exit the nucleus but be recruited to ribosomes only in response to appropriate stimuli.

### Alternative splicing of *ATGSTF11* and *AFC2* genes

Glutathione-S-Transferases (GSTs) are a superfamily that play a prominent role in glutathione (GSH) metabolism of many living organisms [24]. The genome of *Arabidopsis thaliana* encodes 54 functional GSTs, classified into seven clades [25]. Gene *ATGSTF11* (*AT3G03190*) encodes a glutathione S-transferase belonging to the phi class and has been proposed to be involved in the biosynthesis of glucosinolates [26]. Only one isoform is reported in the TAIR10 annotation. Our total RNA-seq analysis assembled three splice variants with an FPKM (Fragments Per Kilobase of transcripts per Million mapped reads) value > = 1 and an isoform expression level > = 3% of the total locus expression. The first isoform in the total sample is identical in structure to the TAIR10 annotated gene model: three constitutive exons are detected. The other two transcripts show the retention of introns 1 and 2. However, only one transcript is generated in polyribosomal RNA-seq assembly, whose structure is consistent with TAIR10 gene model but possesses longer UTRs. Read Alignment analysis confirms that intron retention events are present in total RNA sample but absent from the polyribosomal sample. This comparison between total and polyribosomal RNA-seq analysis suggests that the two isoforms with retained introns in total RNA sample are likely pre-mRNAs that were incompletely spliced.

**Fig 3:**
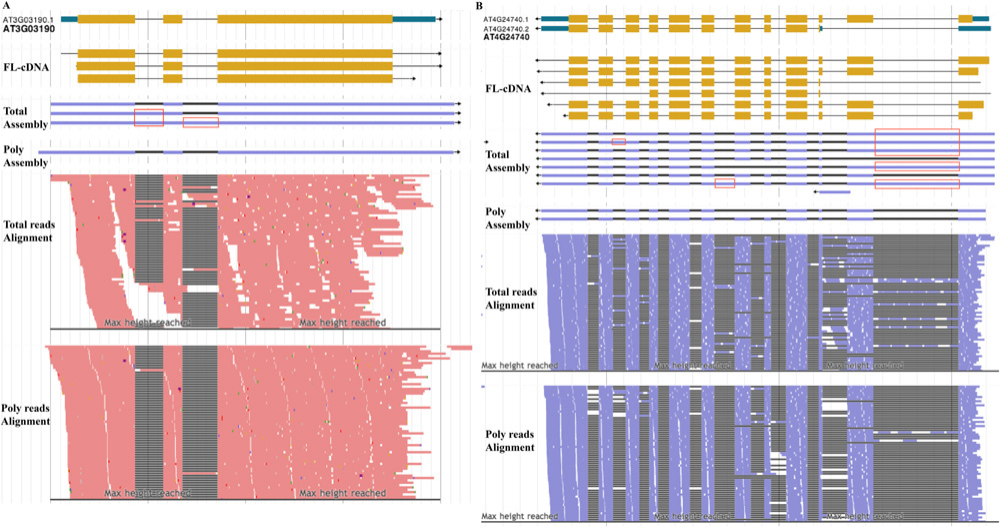
Alternative splicing of *ATGSTF11* (A) and *AFC2* (B) genes. TAIR10 gene models, Full-length cDNA (FL-cDNA), transcripts assembled and reads alignments in the total RNA-seq and polyribosomal RNA-seq datasets are listed from top to bottom. Retained introns in total RNA-seq are highlighted using red rectangles. The red and blue colors represent forward and reverse reads in the read-alignment part, respectively.

To further demonstrate the difference of alternative splicing patterns between total and polyribosomal RNA-seq, we investigated AS events of the gene *AFC2* (*AT4G24740*), which had been analyzed previously [3]. *AFC2* belongs to Clk/STY (LAMMER-type) protein kinases, which play important roles in the regulation of alternative splicing through phosphorylation and interaction with serine/arginine-rich (SR) proteins [27]. Previous analysis of the Arabidopsis transcriptome using RNA-seq technology identified 10 new putative assembled transcripts for *AFC2* [3]. Similarly, our total RNA-seq analysis resulted in seven new assembled isoforms with FPKM value > = 1 and isoform product percentage > = 3%. We compared our assemblies to the TAIR reference gene model (*AT4G24740*). Two types of AS events were observed in the total RNA sample, including intron retention and exon skipping (Fig. 3B). By contrast, the polyribosomal RNA data only detected two isoforms with only two exon-skipping events observed. These results showed that in the total RNA sample *AFC2* retained more introns than in the polyribosomal RNA sample, and that most of the retained introns tend to be spliced in mature mRNA.

## Methods

### Data access and reads alignment

The reference genome, annotation and sequenced full-length cDNAs of *Arabidopsis thaliana* were downloaded from Phytozome (http://www.phytozome.net/). Total and polyribosomal RNA-seq data were obtained from NCBI (Accessions: SRX152606, ERS374056, ERS374057 and ERS374058). For random selection of reads, 95.5 million reads were obtained from the 1st read in the file to the 95.5 millionth read.

Tophat v2.0.8 [18] was implemented to map single-end reads to the TAIR10 genome with a maximum of two mismatches. Minimum and maximum intron lengths were fixed at 60 and 6000 nt, respectively. The rest of the parameters were left as default. Read coverage along transcription units was obtained using annotated gene models in TAIR10. If more than two gene models were annotated for a particular gene, we chose the longest CDS as representative of the gene. The start and the end of each transcription unit were normalized from 0% to 100%, respectively.

### Reconstruction of comprehensive transcript databases

To obtain accurate assembly of transcripts, we applied a well-developed framework, Trinity v20140413 [19] and PASA v20130605 [21], to reconstruct transcripts for total RNA-seq and polyribosomal RNA-seq, separately. For the Trinity *de novo* and genome-guided assembly, we used the default parameters. For building comprehensive transcriptome in PASA, we fixed the maximum intron length to be 5,000 nt, which is similar to a previous study [3].

This pipeline first performs *de novo* assembly using the Trinity *de novo* algorithm, which is a compilation of three distinct sub-programs. After that, we utilized a new feature, implemented in the Trinity package, to perform genome-guided assembly. This algorithm directly applies GSNAP to generate the read alignments, and then partitions the genome-aligned reads into subsets that will each be targeted for independent Trinity *de novo* assembly.

After assembly, transcript abundance estimation was performed using RSEM [28], supported by Trinity. To avoid low abundance (spurious) transcripts, putative isoforms were discarded if their FPKM value was less than 1 and their expression level was less than 3% of total FPKM for that locus. PASA was used to integrate the *de novo* and genome-guided assembly results and built comprehensive transcriptome datasets for the total and polyribosomal RNA-seq.

## Supporting Information

S1 Table Description of total RNA-seq and polyribosomal RNA-seq libraries. (DOCX)
