# Supplementary material for: Polyribosomal RNA-Seq Reveals the Decreased Complexity and Diversity of the Arabidopsis Translatome
Source: PLoS One. 2015 Feb 23;10(2):e0117699. doi: 10.1371/journal.pone.0117699 (PMC4338112; doi:10.1371/journal.pone.0117699)
Supplement: S1 Table — (DOCX) [file pone.0117699.s001.docx]

Table S1. Description of total RNA-seq and polyribosomal RNA-seq libraries

| Data Set | Accession | Material | Age | Seq Platform | Read length | Read Numbers |
| --- | --- | --- | --- | --- | --- | --- |
| Total RNA-seq | SRX152606 | Col0 leaves | 2-3 weeks | Hiseq 2000 | 101 bp | 95.5 million* |
| Poly RNA-seq | ERS374056  ERS374057  ERS374058 | Col0 leaves | 3 weeks | Hiseq 2000 | 101 bp | 95.5 million |

*To achieve a nonbiased analysis, we extracted the same numbers of reads from the top of the total RNA-seq data.
